# 3D Low-Cost Equipment for Automated Peritoneal Dialysis Therapy

**DOI:** 10.3390/healthcare10030564

**Published:** 2022-03-17

**Authors:** Samuel Rivero-Urzua, Juan Carlos Paredes-Rojas, Sergio Rodrigo Méndez-García, Fernando Eli Ortiz-Hernández, Armando Oropeza-Osornio, Christopher René Torres-SanMiguel

**Affiliations:** 1 Instituto Politécnico Nacional, Escuela Superior de Ingeniería Mecánica y Eléctrica Unidad Zacatenco, Sección de Estudios de Posgrado e Investigación, Ciudad de Mexico 07738, Mexico; sriverou0900@alumno.ipn.mx (S.R.-U.); smendezg1401@alumno.ipn.mx (S.R.M.-G.); 2 Instituto Politécnico Nacional, Centro Mexicano para la Producción más Limpia, Acueducto de Guadalupe S/N, La laguna Ticomán, Ciudad de Mexico 07340, Mexico; jparedes@ipn.mx; 3 Instituto Politécnico Nacional, Escuela Superior de Ingeniería Mecánica y Eléctrica, Unidad Culhuacán, Ciudad de Mexico 04260, Mexico; fortizh@ipn.mx; 4 Instituto Politécnico Nacional, Escuela Superior de Ingeniería Mecánica y Eléctrica, Unidad Ticomán, Ciudad de Mexico 07340, Mexico; aoropeza@ipn.mx

**Keywords:** biomechanics, chronic renal disease, automated peritoneal dialysis, peristaltic pump

## Abstract

A breakthrough in peritoneal dialysis (PD) therapy occurred in 1977 with the development of continuous ambulatory peritoneal dialysis (CAPD). Its simplicity, low cost, and ease with which CAPD could be performed on patients at home contributed to the popularity of this procedure. However, there is a need for continuous improvement in building optimal systems for incident chronic kidney disease (CKD) patients. This research showed the design and construction of a simplified prototype of low-cost automated peritoneal dialysis (APD) equipment that meets international standards to automatically regulate infusion and fluid drainage in and out of a patient with low margins of error. Experimental tests allowed the adjustment of the RPM values concerning the flow rate provided. In addition, thanks to the pressure sensor, it was possible to observe a fluctuation ranging from 9 to 13 kPa, which is within the permissible average specified in the catalogs of medical instruments and equipment. Furthermore, a turbidity sensor was added to decrease the possibility of presenting peritonitis. The results showed absolute values of flow, angular velocity, and pressure that it could deliver for use in APD therapies. Finally, the construction of the APD equipment is presented generally, showing the electronic and mechanical components that constitute it.

## 1. Introduction

In Mexico, the number of people with chronic kidney disease (CKD) has increased in the last few years. According to the Global Burden Disease, there was an increment between 1990 and 2015 caused by CKD [1]. The most common ailments are diabetes, arterial hypertension, obesity, and population aging [2,3]. The degenerative evolution for CKD is classified according to the decrease in GFR in five states (Table 1). According to the National Kidney Foundation, Kidney Disease Outcomes Quality Initiative (K/DOQI), those filtration rates that are below 15 mL/min by surface area represent the most severe conditions and establish the requirement for treatment of renal replacement (RRT) [4].

There are two treatments for patients with chronic renal failure: kidney transplant or dialysis. The second has two variants, hemodialysis (HD) and peritoneal dialysis (PD). The objective of both procedures is the same; eliminate the uremic toxins with different molecular weights in the blood [5,6]. However, the performance for each therapy is different; data collected by institutions such as the Mexican Institute of Social Security (IMMS) and the Institute of Security and Social Services for State Workers (ISSSTE) indicate that HD allocates 52.6% to costs per treatment and 39% to complications. In comparison, PD allocates 54.6% of the total cost of care to complications and 36.3% to supplies (bag, cassette, line change) [7]. However, peritoneal dialysis demands a therapy with a wide margin of improvement. In that sense, adjustments to the design of the medical equipment known as a cycler are constantly being developed. A machine for APD administers and prepares the optimal conditions for the dialysis fluid for delivery to reach to the peritoneal cavity. Each therapy performs three work cycles, infusion, permanence, and drain. The pumps transfer dialysate fluid at 35 °C to the peritoneal cavity during the infusion without overfilling. There is an exchange between the blood and the dialyzing solution during the permanence. In addition, through the pump, the main dosing bag is replenished to raise its temperature later. Ultrafiltration (metabolic waste) and infused fluid are removed from the peritoneum in the drainage. Throughout the cycle, the volumetric precision, temperature, flow, and speed are monitored for any irregularity. This is indicated with alarms. Therefore, it is important to select each device that composes the machine [8,9].

The monitoring and performance of the therapy are more efficient in most medical devices making changes to the dosing system. That seeks to modify microfluidic cartridges known as cassettes and use different actuators. Its implementation can be through pneumatic or mechanical pumps. The transfer of the fluid is carried out throughout the cassette with a specific volume over a membrane that generates negative pressures to absorb fluid that the transmission of positive pressure will expel; in that sense, the expansion and compression of the diaphragms give oscillations to the flow; to muffle this fluctuation, it is common to use double-acting pistons [10]. The patent classified as US8221529B2, “Dialysis systems and methods including cassette with air removal”, describes the process for transporting the fluid and the raw material necessary to make the various chambers and diaphragms (sheets) that make up the cartridge; however, this device increases the number of disposable accessories and generates a negative impact on the environment [11]. The patent classified with the number ES2604345T3, named “Infusion pumps and cassettes”, dispenses with the microfluidic cartridge. Instead, it makes a cassette that delivers energy to the system through a peristaltic pump with few elements and directs the flow of the therapy when entering through a flexible conduit; the main character is the transport of immaculate fluid, which is monitored depending on the volume enabled by the conduit. This fluid system contains components that facilitate design and construction. However, it offers an unstable volume, and its monitoring is complex [12]. On the other hand, the creation of cassettes as essential devices for exchanging dialyzing fluids is in patent US8784359B2, called “Cassette system for a peritoneal dialysis machine”, which details their constitution and their installation in a peritoneal dialysis machine. [13]. Likewise, patents that describe the operation of a cassette with different types of activation, either a linear or mechanical motor in patent MXPA05006867A, called “Systems, methods, and apparatus for pumping cartridge-based therapies”, describe systems for fluid supply using a pump cartridge [14]. This type of device, a fastening system, is also required to allow the elements involved in APD to be kept in position. Patent US4585436A, called “Peritoneal dialysis apparatus”, details this characteristic [15]. In addition, patents for devices that use peristaltic pumps or describe their operation and design as in patent WO2013175115A1 called “Linear peristaltic pump” allow the understanding of the main advantages for which it is an excellent way to solve the waste generated by the cassettes and reduce them to a great extent [16,17,18]. It is known that peritoneal dialysis presents complications most of the time because of hygiene during therapy. Despite this, the consequences depend on the kidney damage’s advance. For this reason, it is appropriate to reduce unhealthy food on time to avoid this sickness [19,20]. For the therapy, it has been mentioned that there are different ways to develop it. One of the most popular is the twin-bag, where the exchanges are the principal advantages. However, this one is not expected to suffer complications [21].

For this reason, actions to prevent ailments such as peritonitis have been implemented to reduce the probabilities of existence. This can be detected more efficiently with an analysis of the turbidity [22]. With the advances in technology, developed devices have sensed these properties. It can be measured in line to avoid the extra costs or the interruption of the processes [23]. Peritonitis is known as inflammation of the peritoneal cavity. The fluid inside is affected too and modifies the turbidity, so it can be measured to detect this anomaly and take actions to prevent the advance [24]. It can be detected if there are filtrations by an abscess or something else that was not present at that time or if a perforation in some part of the intestines was made when the peritoneal dialysis was practiced [25,26,27]. If the peritonitis is not detected on time, it can cause severe complications such as bacterial or fungal peritonitis [28]. Some studies have remarked the mortality of this peritonitis, although it is not as common. It can be a severe problem for patients if it is not detected with technologies or medical points of view [29,30].

This research aims to reduce the flow and pressure variations in the delivery of dialytic fluids and redesign the cassette based on an arrangement of pumps. In addition, the measure of the flow and the turbidity also allows the avoidance of an ailment that should transform into peritonitis. This article is organized as follows: The Materials and Methods section explains the machine’s subsystems, their principal characteristics, and the calculus required for the peristaltic pumps’ design. The Results section displays the experimental and theoretical findings that the machine had during the tests. In conclusion, the improvements are presented to optimize the Automated Peritoneal dialysis machine.

## 2. Materials and Methods

**Figure 1:**
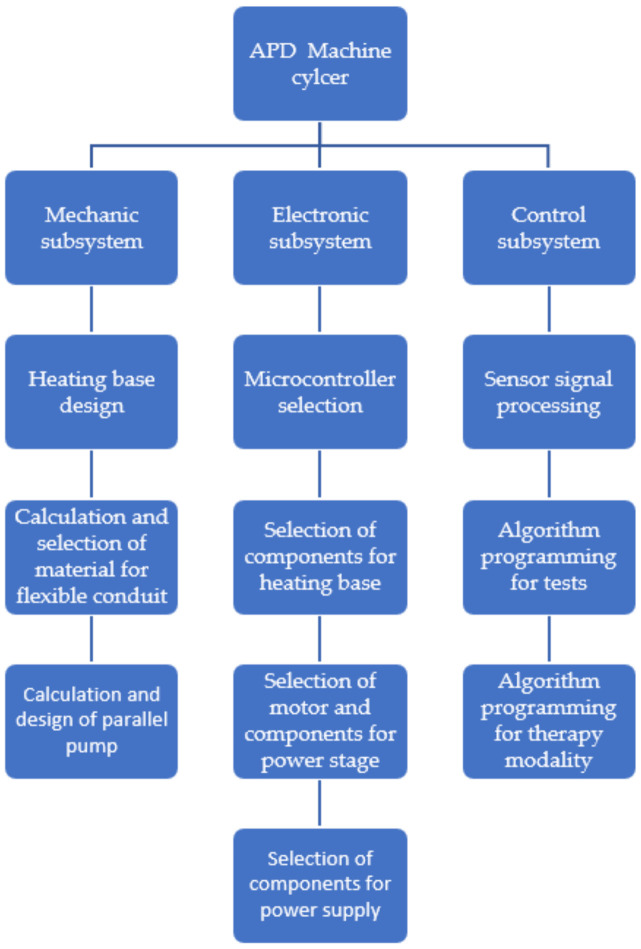
Stages of the machine.

The construction of the machine is divided into three main parts. In the first instance, the mechanical stage lets one know which systems will be activated during the automated peritoneal dialysis treatment, as is shown in Figure 1.

### 2.1. Mechanic Subsystem

The machine’s structure within the mechanical system allows the support of the elements within it.

**Figure 2:**
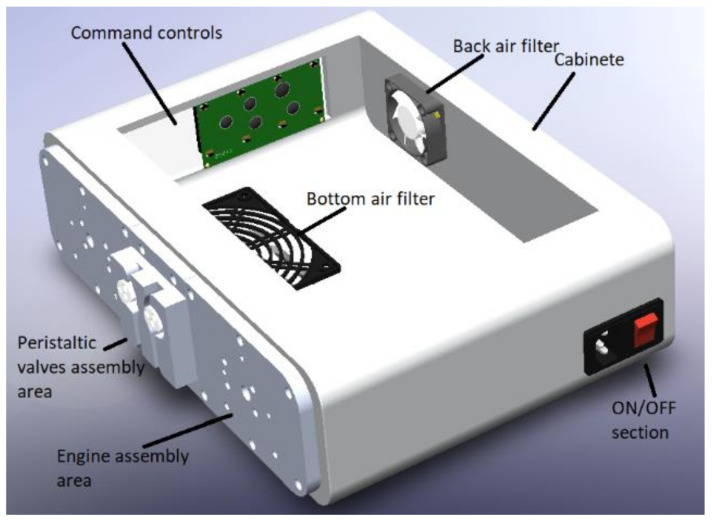
Machine assembly cabinet.

The cabinet of the APD machine is set up with different parts shown in Figure 2; the cabinet supports all the elements, followed by the power supply section of the machine, which is connected through a power cable NEMA 15 splitter. Next, there is an air filter at the back of the machine, which will allow airflow through the machine without introducing unwanted elements. Then, at the bottom, there is another machine’s air filter. The LCD screen and the machine’s controller are on the left side. Next, there are the plates on which the motors and the peristaltic pumps are mounted, and finally, the valves are in the cabinet directly.

#### 2.1.1. Heating Base Design

**Figure 3:**
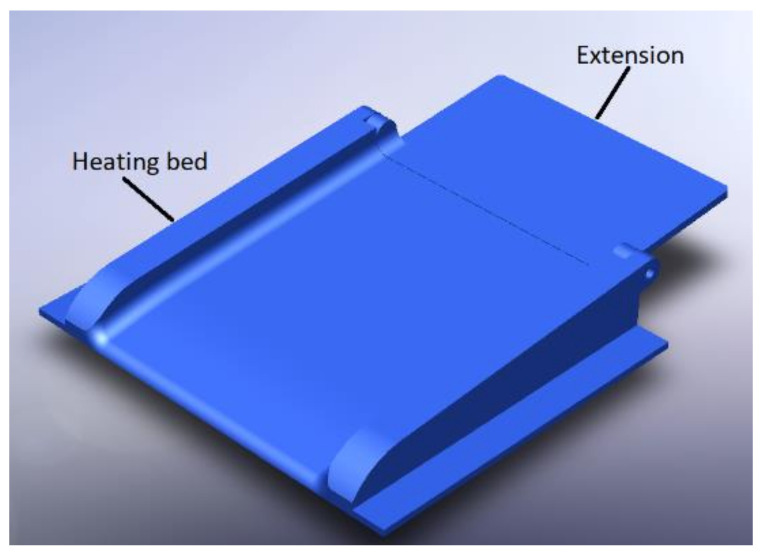
Heating base with extension.

Regarding the construction of the base, it is necessary to know the parameters with which the APD machine will work. In this case, it is usually required that the dialysate liquid is at a temperature of 35 °C, and this temperature must be maintained to avoid any complications, as shown in Figure 3.

The heating base is composed of the section of the thermal bed where the bag of dialyzing liquid rests, which is made up of a material that allows the transmission of heat through it, and it can support bags of more than 2 L. An extension is added for this.

#### 2.1.2. Material for Flexible Duct

For the selection of the flexible hose, the flow rate and the permissible error values must be considered not to damage the patients during the treatment, the pressure delivered by the peristaltic pump must be approximately ±10 kPa, and the flow rate must be $1\frac{mL}{s}$ for drainage and $2\frac{mL}{s}$ for infusion. Typically, Norprene^®^ (Santa Clarita, CA, USA), Tygon^®^ (Santa Clarita, CA, USA), and Marprene^®^ (Santa Clarita, CA, USA) silicone rubber materials are selected. This type of material reduces the risks of tears and ruptures.

#### 2.1.3. Calculation and Design of the Peristaltic Pump

For the fluid flow throughout the circuit, it is established that the flexible conduit is made of medical-grade silicone. The selection of the cross-section is the first data point calculated and evaluated; for this, four theoretical flow simulations ($Q_{Theoretical}$) are carried out, where each study allows the determination of the time a liter of dialysate substance at therapeutic temperature gradually leaves the bag until it is empty; each analysis varies for an outlet of 3, 4, 5, and 6 mm in diameter. The data collected from the theoretical flow are mathematically related to the actual flow ($Q_{Real}$) requested ($2\frac{ml}{s}$), as indicated by Equation (1). The selection reason maintains the premise of bringing the simulated results toward a volumetric efficiency ($\eta_{v}$) close to 80%.

(1) $$\eta_{v}=\frac{Q_{Real}}{Q_{Theoretical}},$$

#### 2.1.4. Calculation and Design of the Parallel Peristaltic Pump

The flow is calculated with Equation (2). This calculation depends on variables such as the angular speed of the selected motor (ω), the volumetric efficiency ($\eta_{v}$), and the volumetric displacement ($D_{v}$). The angular velocity is chosen according to the motor performance and is kept at 80% to 90%. The volumetric efficiency is the data obtained from Equation (1).

(2) $$Q=\omega \cdot \eta_{v}\cdot D_{v},$$

**Figure 4:**
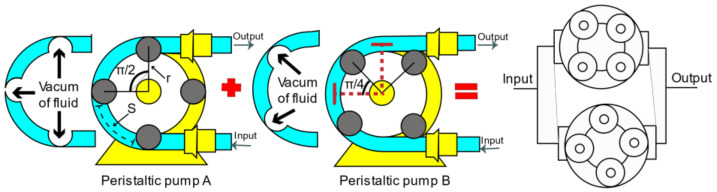
Peristaltic pumps parallel arrangement.

In the analysis for the volumetric displacement described in Equation (3), the number of rollers ($n_{r}$) is defined as four. In the second instance, the arch of the hoses enabled between two rollers is delimited, as shown in Figure 4. Finally, the requested area is calculated with the cross-section of the hose.

(3) $$D_{v}=A\cdot n_{r}\cdot s,$$

where A is the cross-sectional area of the hose and s is the length of the roller measured from the rotor axis.

The design of the parallel peristaltic pump seeks to combine the output pulses generated by the arrangement of 4 separate rollers π2 rad for both pumps; in addition, there is a phase difference of π4 rad between pump A and pump B to reduce flow and pressure fluctuations at the outlet. The flow combination is interpreted with Equation (4).

(4) $$Q_{Outlet}=Q_{A}+Q_{B}=Q_{Outlet},$$

Figure 4 shows the union of two peristaltic pumps on the same axis. Eight stainless-steel rollers are used for configuration, shaft/motor coupling, and a 3D-printed structure. On the other hand, the external structure composed of housings and hardware requires rigidity with a few components to meet the installation, operation, and maintenance requirements. 

**Figure 5:**
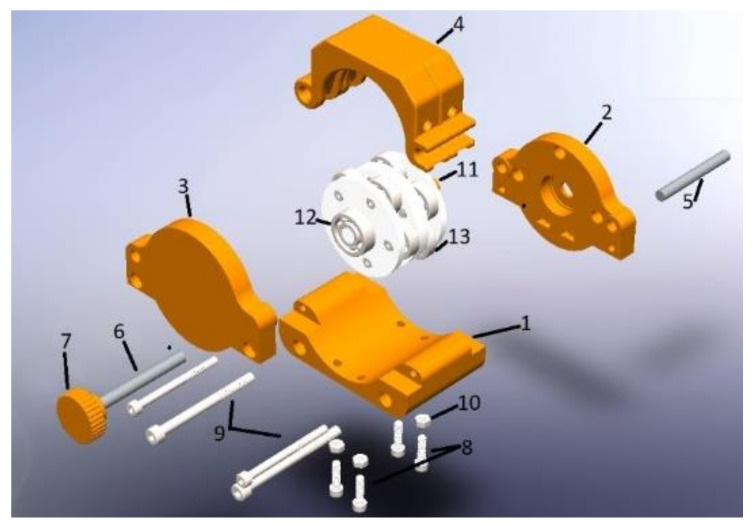
Peristaltic pump structure: (1) Lower casing, (2) Clamping, (3) Back casing, (4) Hose holder housing, (5) Hinge axis, (6) Safety bolt axis, (7) Safety bolt, (8) Socket head screw M4 × 12, (9) Socket head screw M4 × 16, (10) Nut M4, (11) Union stem, (12) Adjusting bearing, (13) Rollers.

Figure 5 shows the external casing (removable) with access to the flexible conduit, where the first part shows how the rolls make a pressure to generate the vacuum and are located at specific points to transport the fluid and control the volume infused.

### 2.2. Selection of Electronic Components for the Cycler

The first requirement mentioned is to raise the temperature of the dialysate liquid up to the recommended operating range. First, a thermal bed raises the temperature from 0 °C to 100 °C. Then, a thermostat module W1209 stabilizes the bed’s temperature; the liquid temperature inside the bag is monitored with the HT-NTC100K3950 sensor. This device handles a measurement range from −35 °C to 350 °C and is waterproof. For the electronic design of the parallel arrangement of the peristaltic pumps, a Hall effect rotary encoder is used coupled to a JGY-371 gearmotor with a maximum speed of 100 RPM, in addition to the L298N driver to control the rotation.

**Figure 6:**
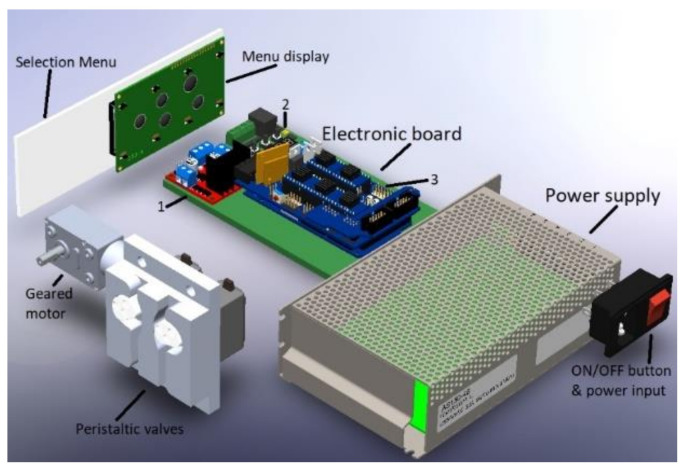
The electronic system of the APD machine: (1) LN298 motor controller, (2) thermostat controller, (3) Arduino board.

To conclude with the circuit, the control part verifies the final conditions of the dialyzer liquid with the set of sensors and a microcontroller that reflects the parameters on an LCD screen. The first variable measured is the flow obtained through a YF-S201 volumetric flow sensor whose characteristics are optimal for the therapy conditions. This device supports temperatures of up to 80 °C and a pressure of 1.75 MPa. Next, monitoring the temperature in the substance requires the HT-NTC100K3950 thermal sensor again. The liquid pressure is verified with a HK3010 sensor located external to the machine to prevent the fluid coming into contact with the sensor. The sensor measures the temperature from the down face of the bag. That supports a magnitude of 10 kPa Lastly, a supply with power capacity is needed for all three stages, shown in Figure 6.

**Figure 7:**
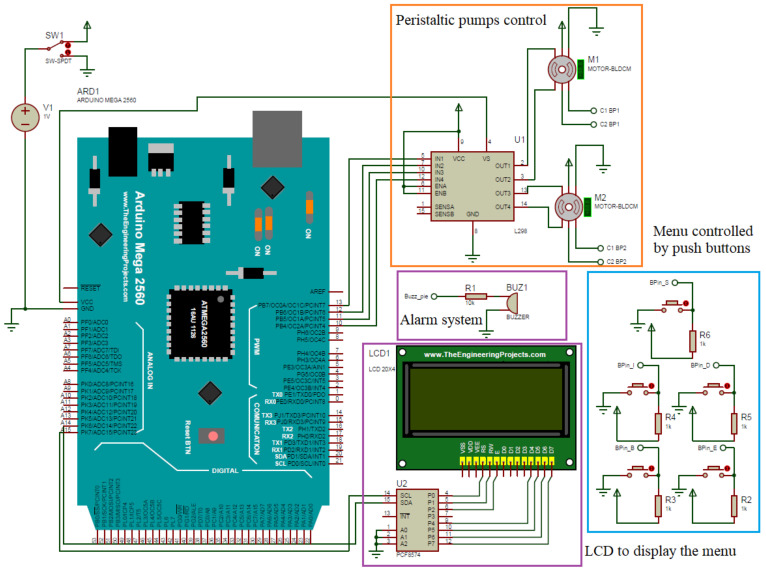
Electronic diagram of the testbed.

Internally, the APD machine consists of electric elements where the Arduino board is mainly located, which has the control of the machine; in turn, for the control of the motors, a L298N circuit is required, which will allow its correct operation, and a thermostat is used that allows the detection of high temperatures in the system in the case of activating and protecting the machine. In addition, the component’s connection determines the distribution of each component and from where it receives the signal command. This circuit is shown in Figure 7.

### 2.3. Control

**Figure 8:**
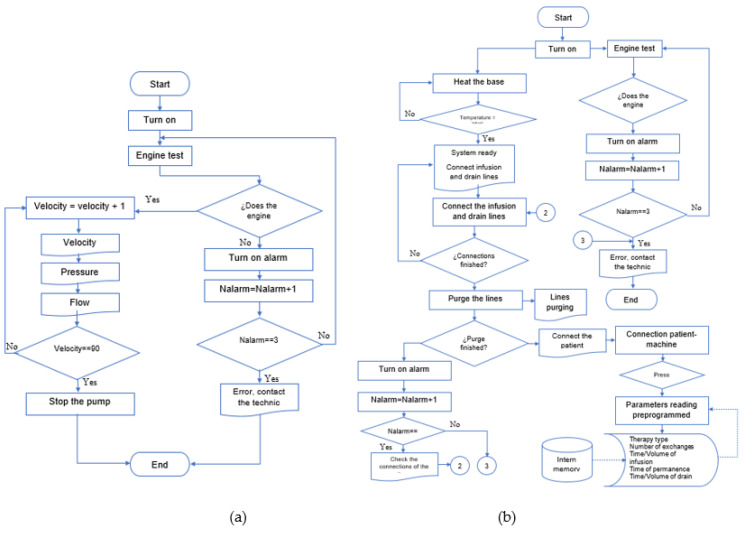
Algorithms’ flowchart: (**a**) electronic diagram in testbed; (**b**) flow diagram in therapy.

The microcontroller is programmed to execute two algorithms, the first for experimental purposes on the actuator with sensor response and the second to perform infusion, permanence, and drain with the ideal operating range (Figure 8). First, in the cassette operation tests, angular velocity variations are made using the pump motor’s pulse width modulation (PWM) technique. Then, the sensors’ signals are processed to be indicated on an LCD screen. Once the tests are concluded, the parallel pump, the priming of the lines, and the therapeutic cycle process are verified. At the same time, the machine conditions are displayed, and the user conditions the therapy process. The information is the measured variables and timers. Figure 8 describes the algorithm for the cycler prototype.

### 2.4. Manufacture of Equipment

**Figure 9:**
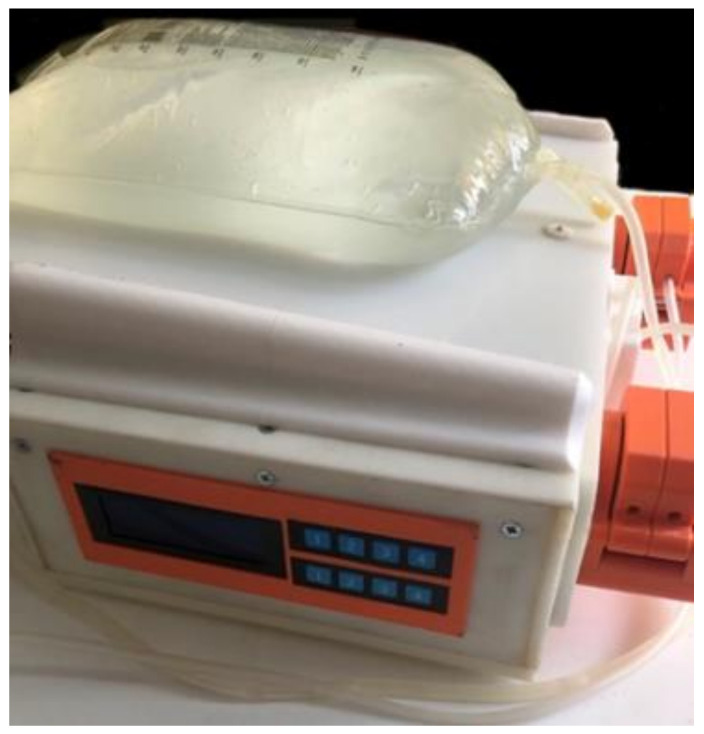
APD prototype.

The design of the heating base is made according to the measurements of the selected electronic plate. An angle of inclination of 18° for the horizontal is considered as a fluid guide in case of damage by the plastic bag; the materials used to condition the base are 3D-printed with PLA filament. The final prototype (Figure 9) is obtained once the tests for each stage have been validated. In addition to looking for a design free to manipulate, another factor that conditions the construction is not exceeding the 21 kg that standardizes this type of machine; the material for the structure is, as with the base, made of PLA filament shown in Figure 9.

### 2.5. Turbidity Measurement

The machine has a turbidity sensor to determine the levels of the drained fluid once it has been on the peritoneum. This is a valuable way to check if there are ailments or possibilities to present peritonitis. According to the medical point of view, if it is at a high level, something else can produce it, so there must be actions to prevent that.

**Figure 10:**
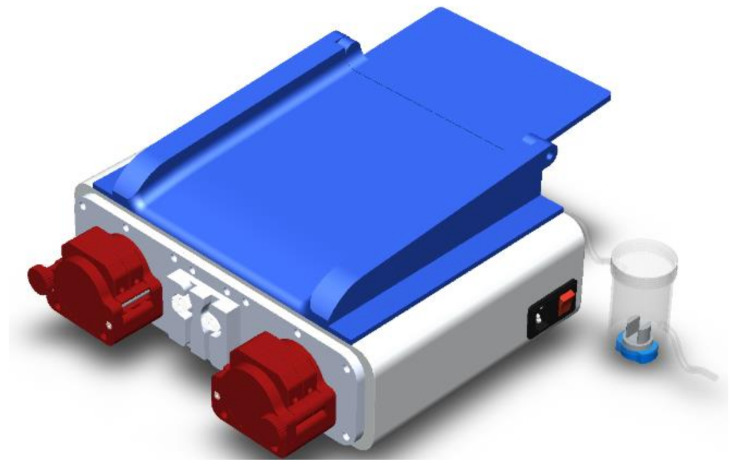
APD machine with turbidity measure section.

Figure 10 shows the section where the device developed to secure a good turbidity measure is located, so there are no perturbations that take incorrect data. It is a simple cylinder with a cover that cleans the sensor, directly contacting the drain fluid. This device is connected to the machine’s output after the flow sensors.

## 3. Results

Once the correct operation of the pump was examined, the heating subsystem was started, and it was verified that the temperature of the dialysate substance rose to the optimal therapy value. Then, two tests were carried out on the control stage. During the first test, the start of the peristaltic arrangement was executed, and the RPM was accelerated uniformly from 0 to 89 RPM to determine the relationship between speed/flow and speed/pressure.

For the second test, the average flow offered at the outlet of the peristaltic parallel arrangement was monitored with a constant speed for 480 s, the average time in which the bag with dialysis substance was emptied. The disposable system was made up of connections, and simple lines replaced the cassette. As a result, there were fewer losses along the circuit. In addition, the temperature, pumping, and control subsystems offered the necessary stability for the fulfillment of the therapy. It was possible to stabilize between 35 °C and 36 °C due to the hysteresis value in the device. The flow results for each diameter proposed in the four case studies are found in Table 2. The combiner flow value represents the flow combination in the parallel pump.

### Monitoring of Therapy Variables

**Figure 11:**
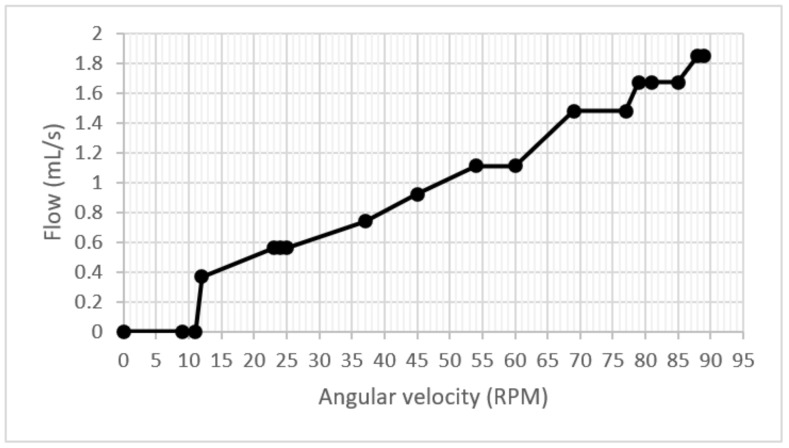
Flow/velocity graph.

The experimental results on the start-up and acceleration test collected the flowmeter’s data, and the graph shows an increasing slope that sometimes remained constant during the increase in speed. One point to highlight is that no drops in flow during the test were observed in Figure 11.

**Figure 12:**
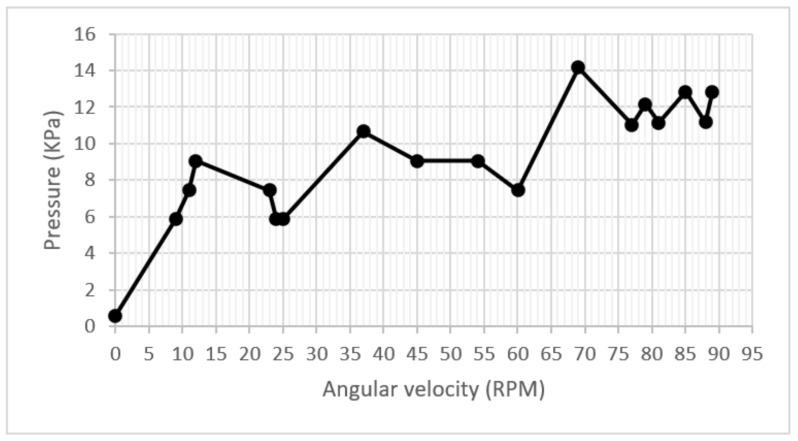
Pressure/velocity graph.

On the same start-up and acceleration test in the pump, a pressure adaptation was shown for each increase in RPM, whose trend increased throughout the test. However, the correspondence rule did not remain constant when fluctuating in the domain of acceleration. During much of the test, the pressure was kept below the maximum allowed value of 12 kPa. The graph showed speeds between 80 and 90 RPM. The pressure fluctuated between 10 and 12 KPa. However, around 64 RPM, 14 KPa obtained a pressure peak. This implies no direct relationship between the increase in speed and pressure, which can be observed in the graph of Figure 12.

**Figure 13:**
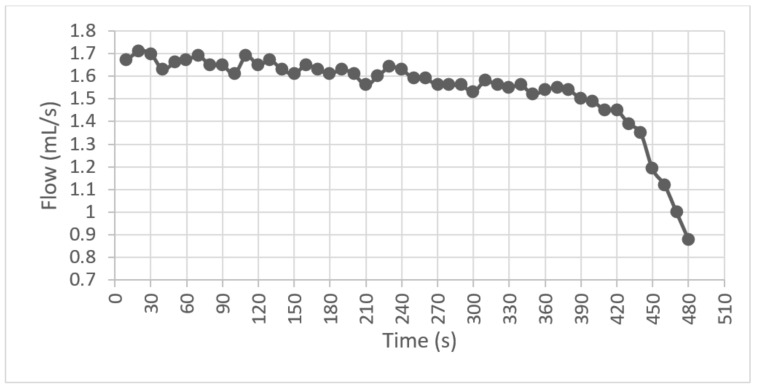
Average flow graph.

For the second test, it was observed that when the speed stabilized at 85 RPM, the average flow delivered was $1.6\frac{mL}{s}$ and the variations were found in a range of $\pm \;0.2\frac{mL}{s}$ during the first 400 s; the bag descended at a capacity of less than 20%, and the average flow started to decrease, as shown in Figure 13.

**Figure 14:**
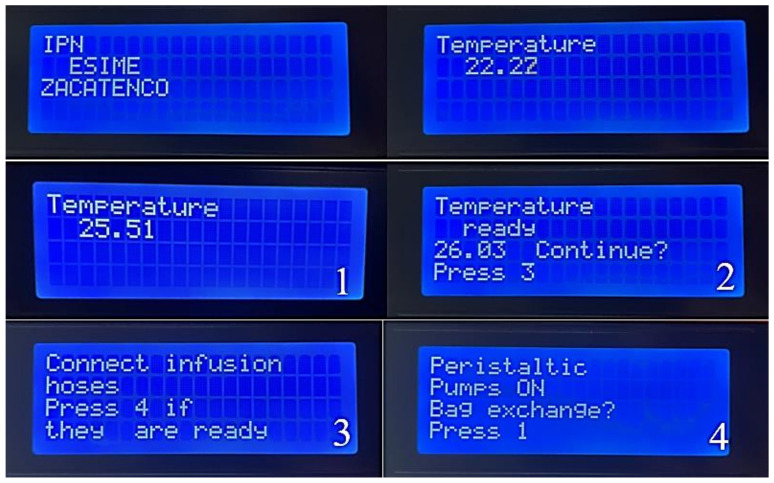
As explained in the last steps, the process menu shows the menu that the machine has while practicing the therapy.

Performance tests were carried out for treatments in which a typical user requires putting the machine into operation by the following steps also shown in the Figure 14.

1. The machine is initialized with the “1” key, and at the same time, the heating bed is activated to decrease the heating time of the peritoneal dialysis bags. Subsequently, the “2” key is activated.
2. Once it reaches the programmed temperature, it goes to the next part of the menu where it shows the temperature that it reached, and later, if it is desired to continue, the next option is selected with the “3” key.
3. The infusion connection hoses are adjusted to the pump’s activation; once they are connected correctly, the switch is flipped on with the “4” key.
4. Finally, if the bag needs to be changed, the reset button is activated, and the process is performed again to verify that it meets the appropriate temperature requirements by pressing the “1” key.

The flow measurement section is a separate section of the machine that only functions to verify that the machine decreases the flow halfway around the desired point. In this way, mostly constant measurements were obtained, ranging between $1.5\frac{mL}{s}$ and $2.5\frac{mL}{s}$.

**Figure 15:**
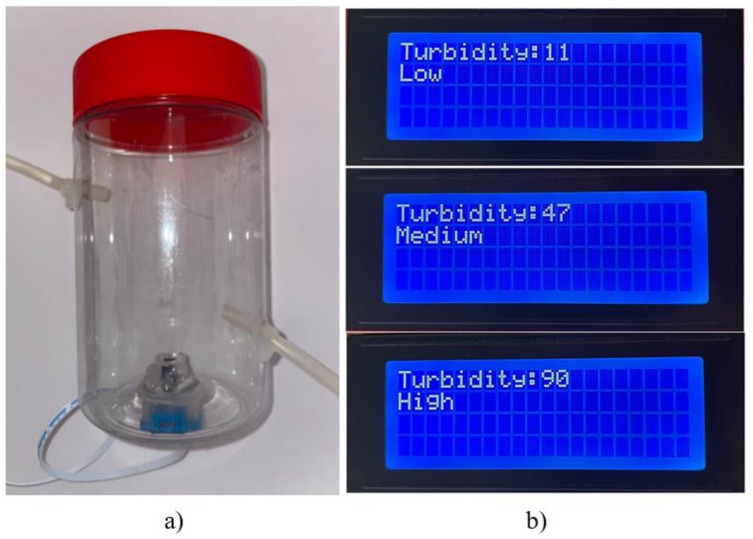
Turbidity measure section: (**a**) experimental sensor assembled, (**b**) display menu.

Table 3 shows the two columns where two flowmeters were installed, one before the peristaltic pump and the other after the pump. It was observed that the inlet flow, despite changes at the outlet, showed the same flow in all the tests carried out. That is, it maintained a constant flow at the outlet. The machine was programmed to identify three turbidity levels as an improvement for the turbidity test. It can be reprogrammed to modify the parameters. This sensor is located at the end of the therapy. While the machine drains fluid from the patient, the turbidity sensor is connected to the drain hoses and measures the turbidity level, as shown in Figure 15.

## 4. Discussion

The machine design seeks to optimize the device by changing how the fluid is transferred. This implies the analysis of the flow transmitter conduit, the implementation of a parallel pump, the reduction in disposable external attachments, and the improvement in the machine’s reliability. However, this prototype considers durability by providing robustness to the rotation and pressure roller mechanism. As a result, there is an increase in weight in the structure, which does not compromise its function. The search for the ideal cross-section through a dynamic study of fluids is a datum that conditions the system’s function. In addition, this flexible conduit is examined based on other variables so that the possible results are directed toward optimizing mechanical resources, but mainly to provide a more enhanced stability in the fluid delivery. The study’s premise is to bring the volumetric efficiency to a value close to 80%.

The voids usually manifest oscillating instability, overpressure, and flow in a conventional cycling machine in the volume that the rollers create when compressing the flexible hose. While, in the proposal of this equipment, the parallel peristaltic pump rotates, pump A generates a volumetric displacement with a volume vacuum generated by a roller, and pump B delivers a total volume enabled between two rollers. The sum of these two volumes decreases the peak values in the fluctuations of the measured variables. An angular velocity between 75 and 85 RPM shows that pressure and flow vary. In addition, the flow does not show drops in its value throughout the test. These results confirm an improvement in stability in fluidic delivery by the prototype, as the patents that exist regarding APD. 

Based on the patents, the leading innovation and improvement presented are the change from operation with disposable cassettes to permanent peristaltic pumps, and it is only necessary to change in case of mechanical failure or any damage that occurs. In addition to the comfortable design, despite being similar to a commercial automated peritoneal dialysis machine, the implementation of peristaltic pumps increases its portability as they are not elements that must be separated or disassembled at some point. Moreover, it is a laborious process as, with the design of a clamping bolt that adjusts the dialysis hoses, it is possible to put it on and take it off easily for hose exchanges if necessary. On the other hand, the machine’s reliability to ensure a correct outflow of the pumps is verifiable with a test bench that allows the reading of these parameters to ensure the desired measurements because precise calculations were made repeatable in the verification processes.

## 5. Conclusions

The objective of the new machine design is to improve the infusion and drainage of dialysate fluid during dialysis therapies, replacing the disposable cassettes with peristaltic pumps that allow the regulation of the flow and ensure that it will be constant.

In addition, extra elements are added to increase the measurement capacity of the machine, either to determine the input and output flows or to detect the turbidity levels of the post-infusion process and prevent risks of peritonitis in patients.

Despite having improvements in the programming and the implementation of mechanisms that allow the therapy to be performed, the use of other devices that further reduce the cost of the machine or even the modification of parameters in different types of therapies is viable to generate a more significant impact and gain presence in the market, thus fulfilling the objective of making treatments available to anyone.

## Figures and Tables

**Table 1:** States for CKD.

State	Description	GFR (mL/min)
-	Increased risk of CKD	60 with risk factors
1	Kidney damage with normal GFR	90
2	Kidney damage with slightly decreased GFR	60–89
3	Moderately decreased GFR	30–59
4	Severely decreased GFR	15–29
5	Kidney failure	<15 or dialysis

**Table 2:** Flow as a function of diameter.

Intern Diameter (mm)	Individual Theoretical Flow (mL/s)	Combined Theoretical Flow(mL/s)
3	0.7632	1.5264
4	1.357	2.714
5	2.1203	4.2406
6	3.0533	6.1066

**Table 3:** Flow measurements.

Test Number	IN Flow (mL/s)	Out Flow (mL/s)
1	11.111	2.222
2	11.111	2.222
3	11.111	2.222
4	11.111	2.222
5	11.111	2.222
6	11.111	2.222
7	8.888	2.222
8	8.888	2.222
9	8.888	2.222
10	8.888	2.222

## Data Availability

Not applicable.
